# Lycorine Attenuates Cardiac Fibrosis Through the Regulation of PYK2 Expression and Activity

**DOI:** 10.1155/cdr/9031925

**Published:** 2026-06-16

**Authors:** Xiuxiu Gao, Ying Zhang, Dongzhe Li, Yufei Chen, Weimiao Qi, Shufang Cui, Chen Wang, Zhong Wang, Quanyi Wang

**Affiliations:** ^1^ Department of Life Science and Technology, China Pharmaceutical University, Nanjing, Jiangsu, China, cpu.edu.cn; ^2^ Jiangsu Key Laboratory for the Research and Utilization of Plant Resources, Institute of Botany Jiangsu Province and Chinese Academy of Sciences (Nanjing Botanical Garden Mem. Sun Yat-Sen), Nanjing, Jiangsu, China

**Keywords:** cardiac fibrosis, ERK1/2, lycorine, PYK2

## Abstract

Lycorine (LYC), the most abundant monomeric compound from *Lycoris radiata* bulbs, possesses a range of biological activities, including antipulmonary fibrosis and cardioprotective effects. However, its role in regulating pathological cardiac remodeling, particularly in cardiac fibrosis, remains unclear. In this study, we used transforming growth factor‐*β*1 (TGF‐*β*1) to stimulate NIH/3T3 cells and primary rat cardiac fibroblasts, which were in vitro models of pathological fibroblast activation, and established a mouse model of cardiac fibrosis via intraperitoneal injection of doxorubicin (DOX). The effects of LYC on TGF‐*β*1‐induced fibroblast activation and migration were assessed using Western blotting, quantitative PCR, scratch assays, and EdU staining. Cardiac damage and fibrosis were evaluated in vivo by echocardiography, enzyme‐linked immunosorbent assay (ELISA), and Masson’s trichrome staining. Through transcriptomic sequencing and bioinformatic analyses, proline‐rich tyrosine kinase 2 (PYK2) was identified as a potential target of LYC. This finding was further validated using siPYK2 and the PYK2 inhibitor PF4618433 in TGF‐*β*1‐induced NIH/3T3 cells. Our results demonstrated that LYC effectively suppressed fibroblast activation, proliferation, and migration. In addition, LYC significantly ameliorated cardiac dysfunction and reduced collagen deposition in myocardial tissues, thereby attenuating the progression of cardiac fibrosis. Mechanistically, these effects were attributed to the regulation of PYK2 expression and activity by LYC. This study provides a scientific basis for understanding the mechanism of LYC in improving cardiac fibrosis and highlights PYK2 as a potential therapeutic target for the treatment of cardiac fibrosis.

## 1. Introduction

Cardiovascular diseases (CVDs) remain a major global health threat and are the leading cause of chronic disease–related mortality, responsible for approximately 18.6 million deaths worldwide in 2019 [1]. In China, the crude incidence rate of CVD among adults aged 18 and above was 620.33 per 100,000 population in 2023, which exhibited a significant upward trend with increasing age [2]. Cardiac fibrosis, a key pathological process in CVDs, is characterized by excessive proliferation and activation of cardiac myofibroblasts and abundant deposition of extracellular matrix (ECM). Myofibroblasts secrete large amounts of cytokines, including *α*‐smooth muscle actin (*α*‐SMA), TGF‐*β*, and collagen (Col) 1, which further promote fibroblast differentiation and lesion expansion [3]. Among various fibrotic pathways, the TGF‐*β* signaling pathway is a well‐established profibrogenic driver, acting through both Smad‐dependent and Smad‐independent mechanisms [4].

Lycorine (LYC), the most abundant monomeric alkaloid extracted from *Lycoris radiata* bulbs, exhibits multiple biological activities. In a hypertensive heart failure model, LYC reduced cardiomyocyte hypertrophy and inflammation by inhibiting the PI3K‐AKT/NF‐*κ*B pathway [5]. In isoprenaline‐induced cardiac insufficiency, LYC downregulated fibrosis‐associated factors via the TGF‐*β*/Sma and mothers against decapentaplegic (Smads) pathway and reduced inflammatory cytokines [6]. In diabetic cardiomyopathy, LYC targeted interleukin enhancer‐binding factor 3 (ILF3) to shift the balance toward Nrf2‐mediated anti‐inflammatory signaling and dampens NF‐*κ*B‐driven proinflammatory responses, further supporting a direct impact on inflammatory pathways and cells [7]. Importantly, a natural compound library screen using human primary cardiac fibroblasts identified LYC as a potential antifibrotic agent [8]. LYC also alleviates DOX‐induced mitochondrial dysfunction and oxidative stress via the silent information regulator (SIRT1)/peroxisome proliferator‐activated receptor *γ* (PPAR*γ*) pathway [9] and attenuates sepsis‐induced myocardial injury by activating AMPK‐related pathways [10]. Despite these findings indicating that LYC has multiple protective effects against CVDs, the role of LYC in pathological cardiac remodeling, particularly cardiac fibrosis, and its underlying molecular mechanisms remain incompletely understood.

Proline‐rich tyrosine kinase 2 (PYK2), a member of the focal adhesion kinase (FAK) family, is activated by extracellular signals such as integrins, angiotensin II, TGF‐*β*, and vascular endothelial growth factor (VEGFA) [11]. Aberrant activation of FAK and PYK2 has been implicated in CVD progression by regulating cell migration, proliferation, and gene expression [12, 13]. Inhibition of FAK/PYK2 attenuates vascular inflammation by reducing the expression of cell adhesion molecules and proinflammatory cytokines [14]. In heart failure, PYK2 impairs intercellular communication by phosphorylating connexin 43 (Cx43), and blocking PYK2 reverses Cx43 remodeling and restores communication [15, 16]. However, whether PYK2 is involved in the antifibrotic effect of LYC in cardiac fibrosis has not been explored.

In this study, we investigated the antifibrotic effects of LYC in both in vitro and in vivo models and elucidated the involvement of the PYK2/ERK signaling pathway, thereby providing a scientific basis for understanding the mechanism of LYC and validating PYK2 as a potential therapeutic target for cardiac fibrosis.

## 2. Materials and Methods

### 2.1. Cell Culture and siRNA Transfection

NIH/3T3 mouse embryonic fibroblasts were obtained from the Chinese Academy of Sciences Stem Cell Bank. Primary rat cardiac fibroblasts were isolated from 0‐ to 3‐day‐old neonatal SD rats. Both cell types were cultured in low‐glucose DMEM supplemented with 10% fetal bovine serum (FBS) and 1% penicillin–streptomycin at 37°C in a 5% CO₂ incubator. Cells were passaged using 0.25% trypsin‐EDTA when they reached 80%–90% confluence.

siRNA targeting mouse PYK2 (si‐PYK2) and negative control siRNA (si‐NC) were synthesized by GenePharma. The confirmed valid sequences of si‐PYK2 # 1 were sense CUACCUGGAACGAAAUAAATT, antisense UUUAUUUCGUUCCAGGUAGTT. si‐NC was a scrambled sequence with no homology to any known gene. NIH/3T3 cells or primary cardiac fibroblasts were seeded into six‐well plates and cultured until 60%–70% confluence. Transfection was performed using siRNA Mate Plus (G04026, GenePharma) reagent according to the manufacturer’s instructions.

### 2.2. Isolation of Primary Rat Cardiac Fibroblasts

Hearts were excised from 0‐ to 3‐day‐old neonatal rats, rinsed in cold Tyrode’s solution, and minced into 1 mm^3^ pieces in a 2‐mL tube. The minced tissue was digested in mixed enzyme solution at 37°C with shaking (180 rpm) for 9 min per cycle; the first digest supernatant was discarded, and five to seven additional digestion cycles were performed. After each cycle, the supernatant was collected into a 15‐mL tube containing 10% FBS low‐glucose medium to stop digestion. The collected cell suspension was centrifuged at 1000 rpm for 8 min, and the pellet was resuspended in low‐glucose complete medium and then filtered through a 200‐mesh sieve. The filtrate was placed in a 6‐cm dish and incubated for 1–1.5 h to allow preferential attachment of fibroblasts. Nonadherent cells (mainly cardiomyocytes) were removed by gently aspirating the supernatant, and the adherent fibroblasts were cultured in low‐glucose medium. When cells reached 80% confluence, they were passaged using standard trypsin‐EDTA.

### 2.3. CCK‐8

Lithophylline hydrochloride LYC (H) was synthesized from MCE and purchased. Cells were cultured to a density of 90% and digested. After 1 × 10^4^ cells per well in a 96‐well plate were cultured for 24 h at 37°C in a 5% CO_2_ incubator, the culture was treated with 10 ng/mL TGF‐*β*1, along with either LYC (H) at concentrations of 0, 1, 2, 5, and 10 *μ*M or PF4618433 (HY‐18312, MCE) at concentrations of 2.5, 5, 10, 20, and 40 *μ*M, and a blank group was set up, with six replicate wells in each group. After 24 h of LYC (H) treatment, 5 *μ*L of CCK‐8 (HY‐K0301, MCE) was added and incubated for 1 h. The absorbance at 450 nm was detected using an enzyme marker, and the data were processed and analyzed.

### 2.4. EdU

Cell proliferation was assessed using an EdU cell proliferation assay kit (BRK0040, ABclonal). Cells at the logarithmic growth phase were seeded into 96‐well plates at an appropriate density. After adherence, they were pretreated with LYC (H) for 12 h, followed by stimulation with TGF‐*β*1 for 24 h. Subsequently, the cells were processed according to the manufacturer’s instructions for the EdU cell proliferation assay kit, and finally, the proportion of positive cells was detected by fluorescence microscopy.

### 2.5. Quantitative Real‐Time PCR

Total RNA from cells or tissues was extracted using TRIzol (15596026CN, Thermo Fisher) according to the instructions. The RNA was then reversed to cDNA using the reverse transcription kit HiScript III RT SuperMix for qPCR (R323‐01, Vazyme), and the cDNA was amplified using ChamQ SYBR qPCR Master Mix (Q311‐02, Vazyme), with 18S as an endogenous control. The primer sequences are shown in Table 1. Finally, the 2^−*ΔΔ*CT^ method was used for statistical analysis.

**Table 1:** qPCR primer sequences.

Name	Primer sequence
Col1a1 (mouse)	Forward: 5 ^′^‐CGATGGATTCCCGTTCGAGT‐3 ^′^
	Reverse: 5 ^′^‐CGATCTCGTTGGATCCCTGG‐3 ^′^
Col3a1 (mouse)	Forward: 5 ^′^‐TGACTGTCCCACGTAAGCAC‐3 ^′^
	Reverse: 5 ^′^‐AGGGCCATAGCTGAACTGAA‐3 ^′^
*α*‐Sma (mouse)	Forward: 5 ^′^‐AGCCATCTTTCATTGGGATGG‐3 ^′^
	Reverse: 5 ^′^‐CCCCTGACAGGACGTTGTTA‐3 ^′^
Mmp9 (mouse)	Forward: 5 ^′^‐GTCCAGACCAAGGGTACAGC‐3 ^′^
	Reverse: 5 ^′^‐ATACAGCGGGTACATGAGCG‐3 ^′^
Fn1 (mouse)	Forward: 5 ^′^‐AGGCAATGGACGCATCAC‐3 ^′^
	Reverse: 5 ^′^‐TTCCTCGGTTGTCCTTCTTG‐3 ^′^
Ccn2 (mouse)	Forward: 5 ^′^‐AGAACTGTGTACGGAGCGTG‐3 ^′^
	Reverse: 5 ^′^‐GTGCACCATCTTTGGCAGTG‐3 ^′^
Col1a1 (rat)	Forward: AGGCATAAAGGGTCATCGTG
	Reverse: ACCGTTGAGTCCATCTTTGC
Col3a1 (rat)	Forward: GAGTTGGAGGTGAAAAGTCTGG
	Reverse: CCTCAGTGTTGATCTTGAAATCC
Fn1 (rat)	Forward: GTACCACTGGCCACACCTAC
	Reverse: TGTCAGCCTGCACGTCCAAC
18S	Forward: 5 ^′^‐CGAACGTCTGCCCTATCAACT‐3 ^′^
	Reverse: 5 ^′^‐CAGACTTGCCCTCCAATGGATCCTCGTT‐3 ^′^

### 2.6. Western Blot

Total proteins of cells and tissues were extracted using RIPA lysate (R0278, Sigma‐Aldrich). Sodium dodecyl sulfate‐polyacrylamide gel electrophoresis (SDS‐PAGE) was performed at 10% and 12.5%. At the end of electrophoresis, the proteins separated on the SDS‐PAGE gels were transferred to a solid phase carrier PVDF (Bio‐Rad) film by wet‐transferring. Five percent skimmed milk was blocked at room temperature for more than 2 h. The proteins were extracted with primary antibodies (Abclonal: anti‐SMA, ab124964, 1:10000; anti‐extracellular signal‐regulated kinase 1/2 [ERK1/2], ab184699, 1:5000, anti‐phosphorylated ERK1/2 [p‐ERK1/2], ab289367, 1:1000; Proteintech: anti‐matrix metalloproteinase‐9 [MMP9], 10375‐2‐AP, 1:2000; Santa Cruz: anti‐GAPDH, sc‐47724, 1:3000; Cell Signaling Technology: anti‐PYK2, 3292S, 1:1000; anti‐phosphorylated PYK2 [P‐PYK2], 3291S, 1:2000) and were incubated at room temperature for 4 h or placed at 4°C overnight. Depending on the species of the primary antibody, select the corresponding mouse secondary antibody or rabbit secondary antibody with a dilution ratio of 1:5000, and incubate at room temperature for 1 h. Mix ECL (E423‐01, Vazyme) Liquid A and Liquid B at 1:1 to prepare the color development solution. Aspirate the color solution to cover the strip. Develop the bands under a chemiluminescence imager, analyze the gray value of the captured bands with ImageJ software, and count the ratio of the target bands to GAPDH using GAPDH as an internal reference.

### 2.7. Wound‐Healing Assay

A black marker was used to draw three horizontal lines at equal distances along the ruler on the back of the six‐well plate, and the cells were inoculated into the six‐well plate and incubated in a 5% CO_2_ incubator at 37°C for 24 h. The control and TGF‐*β*1 groups were pretreated with fresh culture medium, and the treatment group was pretreated with a 5 *μ*M dilution of LYC (H) for 12 h. The cells were drawn a scratch perpendicular to the line of the black marker along the ruler with a yellow tip pressed against the bottom of the plate in each well. PBS was washed once, and serum‐free medium was changed, and TGF‐*β*1 10 ng/mL was given to the TGF‐*β*1 group and the LYC (H) + TGF‐*β*1 treatment group to induce TGF‐*β*1. Immediately after scratching, pictures were taken with an inverted microscope with two fields of view above and below the black horizontal line and six fields of view in each well. Put back in the constant‐temperature incubator to continue incubation; observe the cell migration status at 0, 6, 12, and 24 h; and take pictures at the same position 24 h after scratching.

### 2.8. Cardiac Fibrosis Modeling and Drug Administration in Mice

Seven‐week‐old C57BL/6J male mice (purchased from Jiangsu Huachuang Xinnuo Pharmaceutical Technology Co., Ltd.) were randomly divided into three groups according to body weight (approximately 22–25 g): a control group, a DOX modeling group, and a DOX + LYC (H)‐treated group, with five mice in each group. Mice in the DOX modeling group and the DOX + LYC (H)‐treated group were intraperitoneally injected with DOX (5 mg/kg) once a week for 4 weeks to establish a mouse model of cardiac fibrosis. Starting from the day after the DOX injection, mice in the DOX + LYC (H) treatment group were injected intraperitoneally with LYC (H) solution (5 mg/kg) once every 2 days until the end of the modeling period, while mice in the control group and the DOX modeling group were injected intraperitoneally with 0.9% saline (0.01 mL/g). At the end of Day 29, the mice were subjected to echocardiography, sampling, and subsequent experiments. All experiments on mice were conducted in accordance with the Guidelines for Animal Experiments of the Animal Research Ethics Committee of China Pharmaceutical University. The animal ethics approval number is 2023‐05‐030.

### 2.9. Echocardiography

Turn on the anesthesia machine and set the proportion of isoflurane to 2%–3% to anesthetize the mice. After the mice lost consciousness of voluntary activities, the mice were fixed supine on a 37°C thermostatic plate, and the concentration of isoflurane was adjusted to 1%–2% for continuous anesthesia. The hair on the chest of the mice was wetted with soapy water for hair removal. A small amount of ultrasound coupling agent was applied to the dehairing area, and the probe was gently attached to the top of the coupling agent. The four chambers of the heart were displayed on the screen, which was slowly adjusted until a clear image was obtained, and the cardiograms of 3–5 cardiac cycles were acquired. The collected echocardiograms are analyzed by the software accompanying the small animal ultrasound imaging system, and cardiac function–related indexes such as ejection fraction (EF), shortening fraction, cardiac output (CO), and per‐pulse output can be obtained.

### 2.10. Enzyme‐Linked Immunosorbent Assay

Mice were anesthetized with 2% isoflurane, and blood was collected from the retro‐orbital venous plexus using a capillary glass within 24 h of the fourth intraperitoneal injection of DOX diluent. The samples were allowed to stand at room temperature for 30 min and then centrifuged at 1000 g for 10 min, and the upper layer of yellowish serum was aspirated into new 1.5 mL EP tubes, and 100 *μ*L/tube was dispensed and frozen in an ultra‐low‐temperature refrigerator at −80°C, avoiding repeated freezing and thawing. When it is necessary to test, follow the instructions of the ELISA kit (Beijing 4A Biotech).

### 2.11. Tissue Paraffin Embedding and Sectioning

Dissect the mice and remove the heart tissue after perfusion from the heart with precooled PBS. The removed tissue was rinsed one to two times in PBS, and the heart was gently squeezed with forceps to remove as much blood from the tissue as possible. The upper part of the tissue containing the atria was cross‐sectioned and immersed in 4% paraformaldehyde and fixed for 24 h. Samples were selected according to qPCR and WB results and submitted to AiFang Biological for embedding and sectioning.

### 2.12. Masson’s Trichrome Stain

Masson staining kit was purchased from Servicebio, and the paraffin sections were dewaxed, stained, dehydrated, and sealed according to the manufacturer’s instructions, and then, the slides were scanned with a tissue scanner for a panoramic view of Col fibers in blue and myofibrils, fibrils, and erythrocytes in red. The quantitative analysis of the staining results was performed using ImageJ software.

### 2.13. RNA‐seq

RNA was extracted from NIH/3T3 cells in the control group, TGF‐*β*1‐stimulated group, and LYC (H) + TGF‐*β*1‐treated group, respectively, and the concentration of the samples was measured, and the quality of the samples was examined by the ratios of A260/A280 and A260/A230, and three samples were selected from each group to draw up 5 *μ*L of the samples, and then, the samples were labeled with the sample information on the EP tubes and then sent to Novogene for eukaryotic reference transcriptome sequencing. The samples were labeled on EP tubes and sent to Novogene for eukaryotic transcriptome sequencing.

To identify key regulators of LYC‐mediated antifibrotic effects, RNA‐seq data were analyzed using a standardized filtering pipeline. Differentially expressed genes (DEGs) were first identified using the DESeq2 R package, applying a threshold of |log2 *f* *o* *l* *d* *c* *h* *a* *n* *g* *e*| ≥ 1 and adjusted *p* < 0.05. To capture the dynamic response to treatment, we performed Fuzzy C‐means clustering using the Mfuzz package. Genes were partitioned into six distinct temporal clusters based on their expression profiles across control, TGF‐*β*1, and LYC groups. PYK2 (Ptk2b) was identified as a core member of Cluster 4, which exhibited a characteristic “rescue pattern” (upregulated by TGF‐*β*1 and significantly normalized by LYC). With a high membership score of 0.7998, PYK2 ranked 15th within this functional module.

### 2.14. Statistical Analysis

The data generated in this study were processed and analyzed using Microsoft Excel, ImageJ, and GraphPad Prism, and different statistical methods were adopted for different data types: Student’s *t*‐test was used for the statistical analysis of two independent groups. One‐way ANOVA and the Bonferroni multiple comparison test were used for statistical analysis of multiple treatment comparisons. Statistical significance is indicated by the following numbers:  ^∗^ *p* < 0.05;  ^∗∗^ *p* < 0.01;  ^∗∗∗^ *p* < 0.001;  ^∗∗∗∗^ *p* < 0.0001; and ns represents no statistical difference between the two groups.

## 3. Results

### 3.1. LYC Inhibits the Activation and Migration of Fibroblasts In Vitro

**Figure 1:**
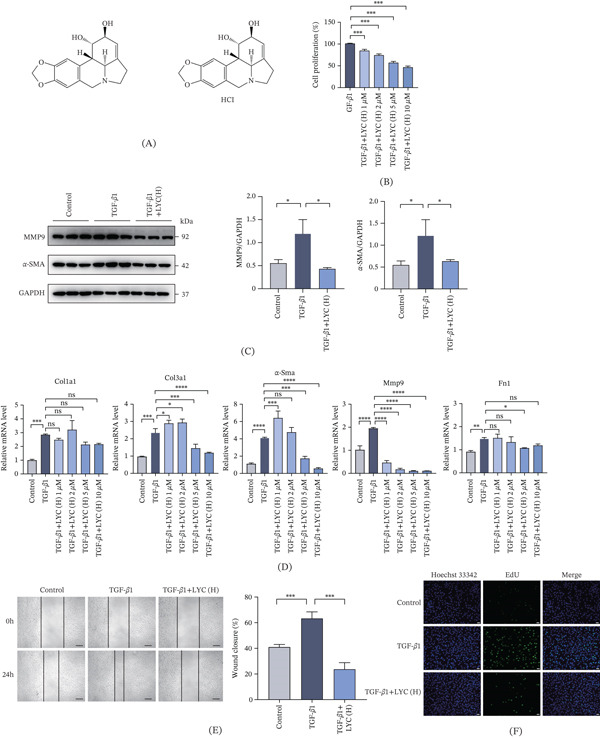
LYC inhibits the activation and migration of fibroblasts in vitro. (A) Structural formulas of LYC and LYC (H). (B) Cell proliferation measured using a CCK‐8 kit, *n* = 3. (C) Western blot for protein expression of MMP9 and *α*‐SMA in NIH/3T3 cells and bar charts, *n* = 3. (D) Real‐time fluorescence qPCR for mRNA expression of Col1a1, Col3a1, *α*‐Sma, Mmp9, and Fn1 in NIH/3T3 cells, *n* = 3. (E) Cell mobility was measured using a scratch test. Scale bar = 250 * μ*m. (F) EdU detection of cell proliferation in each group. Scale bar = 50 * μ*m, *n* = 3.

In order to verify the effect of LYC on cardiac fibrosis in vitro, we used recombinant human TGF‐*β*1 protein to induce activation in NIH/3T3 cells to obtain a cell model of the fibrotic phenotype. We previously found that lithophylline hydrochloride LYC (H) had the same potency as LYC and higher solubility in water, which could avoid the use of solubilization in dimethyl sulfoxide. Therefore, we preferred LYC (H) as an experimental subject. Figure 1A shows the structural formulas of LYC and LYC (H).

Figure 1B shows the results of the CCK‐8 assay. Compared with the TGF‐*β*1 group, LYC (H) inhibited cell proliferation in a concentration‐dependent manner and inhibited 50% of cell proliferation when the concentration of LYC (H) in the treated NIH/3T3 cells reached 10 *μ*M. The optimal concentration of LYC (H) was determined using qPCR to measure the mRNA expression of fibrosis‐related markers. As shown in Figure 1D, TGF‐*β*1 induced an increase in the expression of fibrosis‐related markers such as ColI, Col3a1, *α*‐Sma, Mmp 9, and fibronectin 1 (Fn1) in NIH/3T3 cells at the transcriptional level, which indicated that TGF‐*β*1 induced fibroblast activation in vitro. Meanwhile, the effects of different concentrations of LYC (H) also varied. LYC (H) inhibited the TGF‐*β*1‐induced increase in the mRNA expression of *α*‐Sma and Mmp 9 in a concentration‐dependent manner, and the mRNA expression of Col1a1 tended to decrease (but not significantly). Treatment with low concentrations (1 and 2 *μ*M) of LYC (H) did not have a significant effect on the mRNA expression of Col3a1 or Fn1 and slightly stimulated an increase in Col3a1 transcription, but treatment of cells with LYC (H) at 5 and 10 *μ*M showed a significant inhibitory effect. We concluded that pretreatment with LYC (H) at 5 *μ*M had a general inhibitory effect on the TGF‐*β*1‐induced increase in levels of fibrosis‐related markers. Hence, we used LYC (H) at 5 *μ*M to pretreat cells in subsequent experiments.

Next, we verified the inhibitory effect of LYC (H) at the protein level. As shown in Figure 1C, TGF‐*β*1 induced a consistent increase in the protein expression of *α*‐SMA and MMP9, whereas LYC (H) inhibited the increase in the protein expression of *α*‐SMA and MMP9, which was consistent with qPCR results. Next, we explored if LYC (H) affected the migration of NIH/3T3 cells using a scratch assay. As observed in Figure 1E, at 0 h from scratching, the scratch width and wound area of the three groups were essentially identical, and after 24 h from scratching, the cells in each group migrated to the wound to different degrees. In the control group, TGF‐*β*1 group, and TGF‐*β*1 + LYC (H) 5 *μ*M group, the percent healing of scratches was 40%, 60%, and 20%, respectively. Compared with the control group, TGF‐*β*1 significantly induced cell migration to the scratch site, and LYC (H) + TGF‐*β*1 treatment inhibited cell migration to a greater extent. EdU fluorescence imaging results are shown in Figure 1F. Compared with the control group, the cell proliferation activity in the TGF‐*β*1‐treated group was significantly enhanced, indicating that TGF‐*β*1 markedly promoted cellular DNA synthesis and proliferation. However, after LYC intervention, the number of EdU‐positive cells was significantly reduced compared with the TGF‐*β*1 group, and the green fluorescence signal was notably weakened, with cell proliferation levels returning to near‐control status. These findings collectively indicate that LYC not only reverses the proliferation‐promoting effect induced by TGF‐*β*1 but also inhibits the activation and proliferation of fibroblasts in vitro.

### 3.2. LYC Inhibits the Activation and Migration of Primary Rat Cardiac Fibroblasts In Vitro

**Figure 2:**
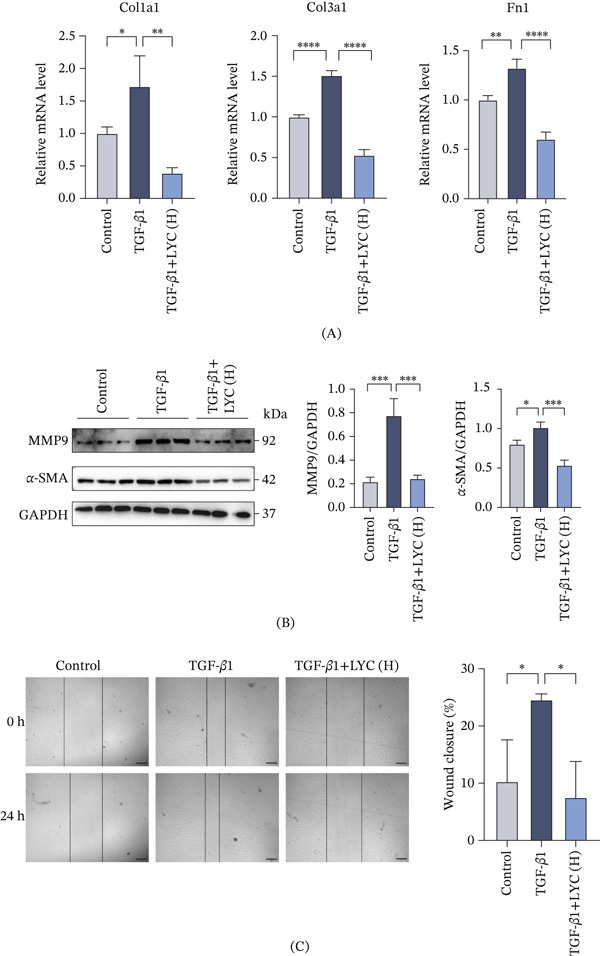
LYC inhibits the activation and migration of primary rat cardiac fibroblasts in vitro. (A) Real‐time fluorescence qPCR for mRNA expression of Col1a1, Col3a1, and Fn1 in primary rat cardiac fibroblasts, *n* = 3. (B) Western blots for protein expression of MMP9 and *α*‐SMA in primary rat cardiac fibroblasts and bar charts, *n* = 3. (C) Cell mobility was measured using a scratch test. Scale bar = 100 * μ*m, *n* = 3.

To investigate the effect of LYC on the activation of primary rat cardiac fibroblasts in vitro, we induced cell activation with TGF‐*β*1 and treated the cells with LYC (H) (5 *μ*M). The experiment was divided into three groups: control, TGF‐*β*1, and TGF‐*β*1 + LYC (H). qPCR results (Figure 2A) showed that TGF‐*β*1 significantly upregulated the mRNA expression levels of the fibrosis markers Col1a1, Col3a1, and Fn1, while LYC (H) treatment markedly suppressed these upregulations. Western blot results (Figure 2B) revealed that the protein levels of *α*‐SMA and MMP9 were significantly increased in the TGF‐*β*1 group compared with the control group. After LYC treatment, the expression levels of both proteins were significantly decreased. The scratch assay results (Figure 2C) showed that TGF‐*β*1 stimulation dramatically enhanced cell migration, with the percentage of wound healing being significantly higher than that of the control group within 24 h; in contrast, LYC (H) treatment significantly inhibited cell migration. These results indicate that LYC inhibits TGF‐*β*1‐induced activation and migration of primary rat cardiac fibroblasts. The results obtained in primary rat cardiac fibroblasts are highly consistent with the findings in NIH/3T3 cells, further confirming the inhibitory effect of LYC on cardiac fibroblast activation and migration, thereby strengthening the clinical relevance of our conclusions.

### 3.3. LYC Ameliorates DOX‐Induced Cardiac Insufficiency in Mice

**Figure 3:**
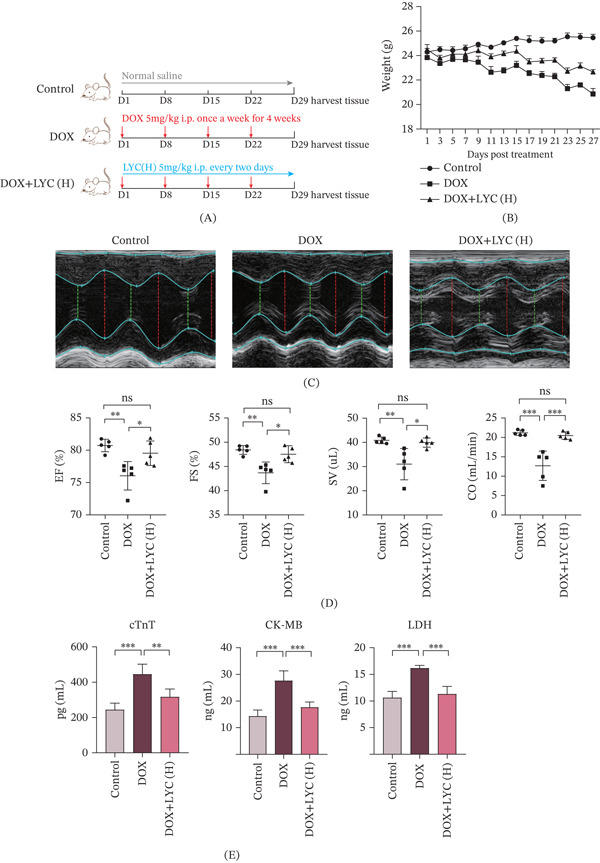
Lycorine improves DOX‐induced cardiac insufficiency in mice. (A) “Roadmap” of mouse modeling. (B) Graph showing bodyweight changes in mice, *n* = 5. (C) Representative echocardiograms of mice. (D) Ejection fraction, shortening fraction, stroke volume per beat, and cardiac output of mice, *n* = 5. (E) Expression of cardiac troponin T, creatine kinase isoenzyme, and lactate dehydrogenase detected by enzyme‐linked immunosorbent assays, *n* = 5.

We used DOX to construct a fibrosis model in mice (Figure 3A). Figure 3B shows the curves for bodyweight change of mice in each group during modeling. The bodyweight of mice in each group was very similar at the beginning of the modeling period. After 4 weeks of modeling, the bodyweight of control mice increased slightly to 26 g, the bodyweight of mice in the DOX modeling group decreased continuously, and the bodyweight of mice in the DOX + LYC (H) treatment group decreased, but the decrease was smaller than that in the DOX modeling group. Hence, LYC partially alleviated the DOX‐induced body weight loss in mice.

We further investigate if LYC (H) could improve the effect of cardiac fibrosis on normal cardiac function. Echocardiography was performed on mice after 4 weeks of modeling to evaluate the cardiac function of mice in each group. The echocardiographic images in Figure 3C reveal that the left‐ventricular end‐diastolic internal diameter and end‐systolic internal diameter of DOX‐modeled mice were significantly shortened. Figure 3D shows that, compared with the control group, the EF, fractional shortening (FS), CO, and stroke volume (SV) of the DOX modeling group were reduced significantly, suggesting that the left‐ventricular systolic function of mice was abnormal and that cardiac insufficiency was present. Treatment with DOX + LYC (H) significantly inhibited the decrease in EF, FS, CO, and SV caused by modeling, with their levels similar to those of the control group. Hence, LYC could improve the cardiac insufficiency caused by cardiac fibrosis.

Cardiac troponin T (cTnT), creatine kinase, mb form (CK‐MB), and lactate dehydrogenase (LDH) are released into serum if myocardial tissues are damaged. CK‐MB and LDH in serum are commonly used as specific markers of myocardial injury. Therefore, after collection of whole blood from the retro‐orbital plexus, we obtained serum within 24 h after the fourth injection of DOX. Then, we measured the concentrations of cTnT, CK‐MB, and LDH in the serum of mice in each group using ELISAs (Figure 3E). The levels of cTnT, CK‐MB, and LDH in the DOX modeling group were increased significantly compared with those of the control group, indicating that the myocardium had been damaged. The levels of cTnT, CK‐MB, and LDH were significantly lower in the DOX + LYC (H) group than in the DOX modeling group, and there was no significant difference from the control group. Hence, LYC could avoid the myocardial injury caused by DOX to a certain extent. Taken together, these results suggest that LYC could improve the cardiac insufficiency and myocardial injury caused by DOX in mice.

### 3.4. LYC Inhibits DOX‐Induced Cardiac Fibrosis

**Figure 4:**
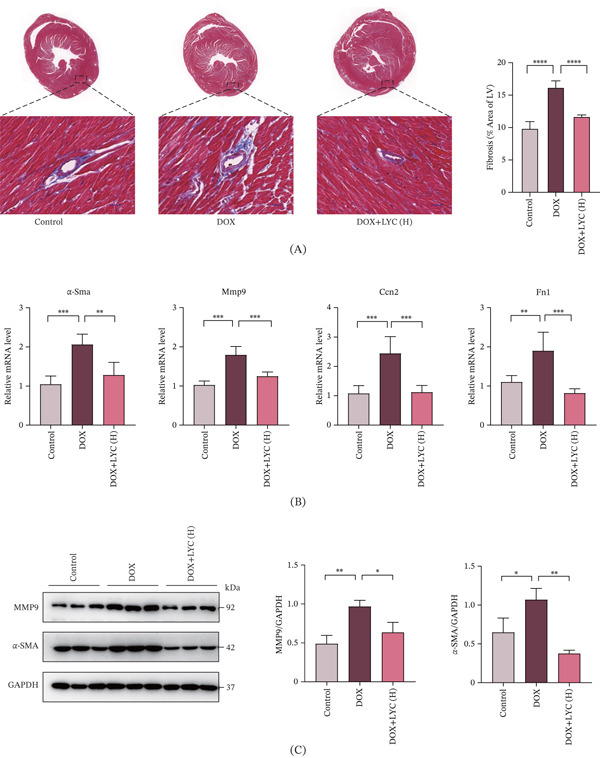
Lycorine inhibits DOX‐induced cardiac fibrosis. (A) Representative staining results (Masson’s trichrome) for cardiac mouse tissue and the corresponding quantitative bar graph, scale bar = 100 * μ*m, *n* = 5. (B) Real‐time fluorescence qPCR for mRNA expression of *α*‐Sma, Mmp9, Ccn2, and Fn1 in mice, *n* = 5. (C) Western blots for protein expression of MMP9 and *α*‐Sma in mice and bar charts, *n* = 5.

Based on the preliminary demonstration that LYC (H) could improve DOX‐induced cardiac insufficiency in mice, we further investigated the effect of LYC (H) on cardiac fibrosis. The cardiac tissues of three groups of mice were fixed, embedded, sectioned, and stained using Masson’s trichrome. Figure 4A shows that, in the control group, the cardiomyocytes of mice were arranged neatly and densely, and a few blue Col fibers were scattered in the interstitium of the myocardium. In the DOX modeling group, cardiomyocytes were lysed, myocardial fibers were disorganized, and many blue Col fibers were deposited in the interstitium of the myocardium and the periphery of blood vessels. After LYC intervention, myocardial Col deposition was significantly reduced, and myofibril structure was improved. Quantitative analysis of Masson staining results showed that, compared with the control group, the percentage of myocardial fibrosis area in the left ventricle was extremely significantly increased in the DOX‐treated group, while that in the DOX + LYC (H) group was extremely significantly decreased compared with the DOX group. These findings indicate that LYC can effectively alleviate DOX‐induced myocardial fibrosis and exert a significant cardioprotective effect.

We examined using qPCR if there were changes in the transcript levels of the cardiac fibrosis‐related markers *α*‐Sma, Mmp9, connective tissue growth factor (Ccn2), and Fn1 among groups. The transcript levels of *α*‐Sma mRNA were twofold higher in the DOX modeling group compared with the control group and were significantly decreased in the DOX + LYC (H) group compared with the DOX model group; the transcript levels of the DOX + LYC (H) group were the same as those of the control group. LYC (H) treatment prevented the transcript levels of these fibrosis markers from increasing, suggesting that LYC (H) inhibited DOX‐induced cardiac fibrosis to a certain extent (Figure 4B).

The left panel of Figure 4C shows a WB strip chromatogram. The expression of *α*‐SMA and MMP9 increased significantly in the DOX modeling group, and LYC (H) treatment inhibited the increase in the protein expression of *α*‐SMA and MMP9. The right panel of Figure 3C shows a WB strip chromatogram. Compared with the control group, the expression of *α*‐SMA and MMP9 in the DOX‐induced cardiac tissues of mice increased by onefold, and LYC (H) treatment prevented the increase in the levels of these fibrotic markers to a certain extent (Figure 4C). The expression of *α*‐SMA and MMP9 in mouse heart tissue was increased by onefold in DOX‐induced mice compared with that in control mice, and the expression of these two marker proteins was reduced significantly after LYC (H) treatment. In conclusion, we revealed that LYC had a protective effect on the heart, could improve DOX‐induced cardiac fibrosis significantly, and had a potential antifibrotic effect.

### 3.5. PYK2 Is Involved in the Regulation of Cardiac Fibrosis

According to the results of transcriptome sequencing returned by Beijing Novozymes, the expression of 911 genes was upregulated significantly after TGF‐*β*1 stimulation compared with that in the control group; the expression of 1161 genes was downregulated significantly after LYC (H) + TGF‐*β*1 treatment compared with that in the TGF‐*β*1 group. In addition, 83 overlapping DEGs were identified, and we plotted a heat map for them (Figure 5A). These DEGs needed to be downregulated further to elicit gene functions. Hence, we analyzed the functional enrichment of these coexpressed DEGs using the GO database and created bubble plots. These genes were significantly enriched in biological processes such as ECM composition, cell growth regulation, angiogenesis, and epithelial cell migration regulation (Figure 5B). Analyses of signaling pathway enrichment using the KEGG database revealed that the downregulated expression of the DEGs after LYC (H) treatment led to enrichment in PI3K‐Akt, calcium signaling, and metabolic pathways (Figure 5C).

Figure 5PYK2 is involved in regulating cardiac fibrosis. (A) Transcriptome sequencing, screening of differentially expressed genes, and heat maps. (B) A total of 83 DEGs were subjected to functional enrichment analyses using the GO database, and bubble plots were drawn. (C) A total of 83 DEGs were analyzed for signaling pathway enrichment using the KEGG database, and bubble maps were drawn. (D) Real‐time fluorescence qPCR for mRNA expression of Trpv2, Vegfa, Itgb3, Pyk2, and Notch4 in NIH/3T3 cells, *n* = 3. (E) Real‐time fluorescence qPCR for mRNA expression of Vegfa and Pyk2 in mice, *n* = 5. (F) Western blots for protein expression of PYK2, p‐PYK2, ERK, and p‐ERK in NIH/3T3 cells and bar charts, *n* = 3. (G) Western blots for protein expression of PYK2, p‐PYK2, ERK, and p‐ERK in mice and bar charts, *n* = 5.

**Figure:**
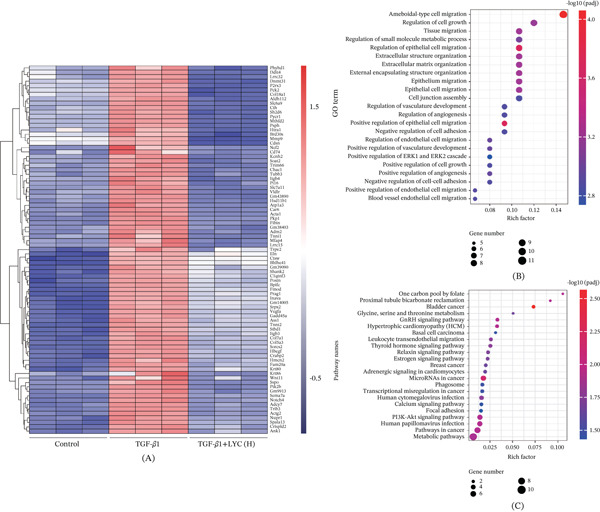
(a)

**Figure:**
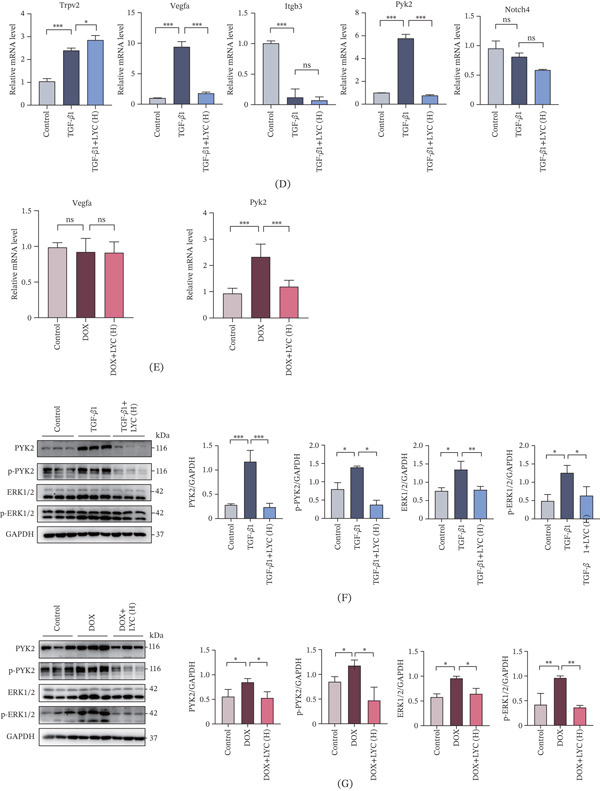
(b)

Combining analyses of functional enrichment (using the GO database) and enrichment of signaling pathways (KEGG database), we initially screened five genes that were closely related to CVD progression: transient receptor potential vanilloid ion channel subtypes 2 (Trpv2), Vegfa, integrin subunit beta 3 (Itgb3), Pyk2, and neurogenic locus notch homolog protein 4 (Notch4). Then, we measured the expression of these five genes at the transcriptional level in NIH/3T3 cell samples to ascertain if the qPCR results were consistent with the trend of changes obtained by RNA‐seq. Figure 5D shows that only the expression of Vegfa and Pyk2 was consistent with our expectation. We performed further validation in the cardiac tissue samples of mice with cardiac fibrosis. qPCR results (Figure 5E) showed that Vegfa expression was close to that of other groups. The mRNA expression of Pyk2 was nearly 1.2‐fold higher in the DOX modeling group than in the control group, and the expression of PYK2 in the DOX + LYC (H) group was comparable with that in the control group. Therefore, Pyk2 expression showed the same trend in cellular and animal samples. Hence, PYK2 could be a target and may play an important part in cardiac fibrosis.

As an NRTK, PYK2 is active only after phosphorylation and is involved in downstream signaling. Therefore, we first measured the protein expression of PYK2 and p‐PYK2 in differently treated cell samples. WB chromatograms suggested that PYK2 and p‐PYK2 showed the same trend of change. That is, the amount of native protein was increased significantly, and the phosphorylation level was increased simultaneously in the TGF‐*β*1 group, whereas LYC (H) pretreatment significantly inhibited the TGF‐*β*1‐induced increase in the expression of native protein and phosphorylated protein (Figure 5F). Similarly, the protein expression of PYK2 and p‐PYK2 was increased in DOX‐induced heart tissues with cardiac fibrosis, and the protein expression of PYK2 and p‐PYK2 was suppressed upon LYC (H) administration (Figure 5G). The trends of changes in the mRNA and protein expression of PYK2 in differently treated NIH/3T3 cell samples or cardiac tissue samples from mice with cardiac fibrosis were consistent and matched with the results of RNA‐seq. The in vivo and in vitro experimental results stated above were highly consistent with the data from biosignature analysis, from which we preliminarily concluded that LYC regulated the development of cardiac fibrosis through PYK2.

Next, we examined the changes in the protein expression of the PYK2 downstream protein ERK1/2 and p‐ERK1/2 simultaneously in cells and cardiac tissue samples of mice with cardiac fibrosis. Figure 5F,G reveals that ERK1/2 and p‐ERK1/2 showed a significant increase in the amount of background protein in the TGF‐*β*1 group or DOX modeling group, along with increased phosphorylation, whereas LYC (H) pretreatment significantly suppressed the increase in the protein expression of ERK1/2 and p‐ERK1/2. The trend of changes in the expression of ERK1/2 and p‐ERK1/2 was consistent with that of PYK2 and p‐PYK2 in all groups, suggesting that PYK2 may regulate cardiac fibrosis through the ERK signaling axis.

### 3.6. Knockdown of PYK2 Suppresses TGF‐*β*1‐Induced Activation of NIH/3T3 Cells

**Figure 6:**
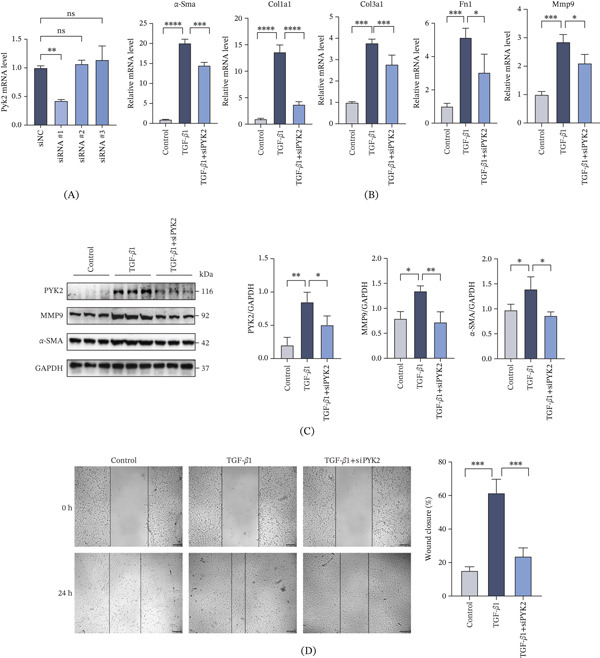
Knockdown of PYK2 suppresses TGF‐*β*1‐induced activation of NIH/3T3 cells. (A) Real‐time fluorescence qPCR detection of the knockdown efficiency of three siRNAs targeting pyk2, *n* = 3. (B) Real‐time fluorescence qPCR for mRNA expression of *α*‐Sma, Col1a1, Col3a1, Fn1, and Mmp9 in NIH/3T3 cells, *n* = 3. (C) Western blots for protein expression of MMP9 and *α*‐SMA in NIH/3T3 cells and bar charts, *n* = 3. (D) Cell mobility was measured using a scratch test. Scale bar = 50 * μ*m, *n* = 3.

To further validate the role of PYK2 in cardiac fibrosis, we knocked down PYK2 in NIH/3T3 cells using specific siRNA. The knockdown efficiency is shown in Figure 6A, where siRNA #1 exhibited the strongest knockdown effect and was therefore selected for subsequent experiments. Cells were divided into three groups: control, TGF‐*β*1, and TGF‐*β*1 + siPYK2. qPCR results (Figure 6B) showed that TGF‐*β*1 significantly upregulated the mRNA expression of the fibrosis markers *α*‐Sma, Col1a1, Col3a1, Fn1, and Mmp9, while PYK2 knockdown markedly suppressed these upregulations. Western blot results (Figure 6C) revealed that the protein levels of MMP9 and *α*‐SMA were significantly increased in the TGF‐*β*1 group compared with the control group, whereas PYK2 knockdown restored these protein levels back to control levels. The scratch assay results (Figure 6D) showed that TGF‐*β*1 stimulation dramatically enhanced cell migration, with cells nearly closing the scratch gap within 24 h; in contrast, PYK2 knockdown significantly inhibited cell migration.

### 3.7. PYK2 Inhibitor PF4618433 Suppresses TGF‐*β*1‐Induced Activation of NIH/3T3 Cells

**Figure 7:**
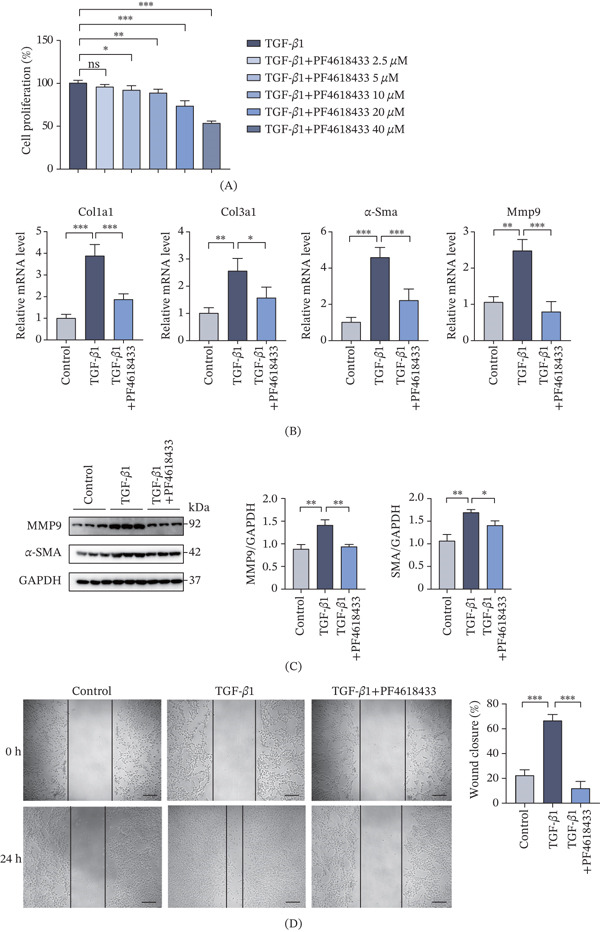
PYK2 inhibitor PF4618433 inhibits TGF‐*β*1‐induced activation of NIH/3T3 cells. (A) Cell proliferation measured using a CCK‐8 kit, *n* = 3. (B) Real‐time fluorescence qPCR for mRNA expression of Col1a1, Col3a1, *α*‐Sma, and Mmp9 in NIH/3T3 cells, *n* = 3. (C) Western blots for protein expression of MMP9 and *α*‐SMA in NIH/3T3 cells and bar charts, *n* = 3. (D) Cell mobility was measured using a scratch test. Scale bar = 250 * μ*m, *n* = 3.

We wished to further confirm the mechanism of cardiac fibrosis regulation by stilbenes. We treated fibroblasts with the PYK2 inhibitor PF4618433 to verify the effect of PYK2 inhibition on cardiac fibrosis. The CCK‐8 assay was used to determine the appropriate treatment time and concentration of PF4618433. Figure 7A shows that PF4618433 inhibited the proliferation of fibroblasts in a concentration‐dependent manner. Cell proliferation was not affected by a low concentration (2.5 *μ*M). As the concentration of PF4618433 increased (5, 10, and 20 *μ*M), the percent inhibition of cell proliferation increased gradually to 10%, 15%, and 25%, respectively. When the concentration of PF4618433 reached 40 *μ*M, it could inhibit the proliferation of 50% of cells. Based on the CCK‐8 assay, we chose to treat cells with 20 *μ*M of PF4618433, which could inhibit cell proliferation significantly. PF4618433 was added in the form of fluid exchange, and after 3 h of pretreatment, TGF‐*β*1 was added to cotreat the cells. After 24 h of induction, cells were collected, and RNA was extracted. Figure 7B shows the qPCR results. The mRNA expression of the fibrosis markers Col1a1, Col3a1, *α*‐Sma, and Mmp9 was increased significantly under induction by TGF‐*β*1, and treatment with PF4618433 suppressed the increase in the expression of these profibroblast genes at the transcription level. Figure 7C reveals that the WB results had the same trend as qPCR data, with the protein expression of MMP9 and *α*‐SMA in the TGF‐*β*1 group being 1.5 times higher than that in the control group, and this increase was restored to control levels by PF4618433 treatment. A scratch assay showed that PF4618433 pretreatment inhibited cell proliferation (Figure 7D). After 24 h of TGF‐*β*1 induction, cells from the TGF‐*β*1 group migrated toward and almost occupied the scratch, whereas PF4618433 pretreatment significantly inhibited the migration of cells toward the scratch, with no significant movement compared with the scratch at 0 h. The results from the CCK‐8 assay, qPCR, WB, and scratch assay were highly consistent with each other. We believe that LYC most likely regulates cardiac fibrosis through PYK2. Collectively, the pharmacological PYK2 inhibitor PF4618433 recapitulated the antifibrotic effects observed with knockdown of PYK2, further confirming that PYK2 mediates TGF‐*β*1‐induced fibroblast activation.

## 4. Discussion

Cardiac fibrosis is a key factor causing heart failure and arrhythmia. Efficacious drugs that can reverse cardiac fibrosis are lacking. To date, no effective drugs are available to reverse cardiac fibrosis. Some drugs used in the treatment of heart failure and hypertension have emerged as candidates in antifibrotic therapy. For example, angiotensin receptor antagonists and inhibitors of angiotensin‐converting enzymes are used to slow the progression of cardiac fibrosis. However, these drugs are used mainly for cardiac fibrosis caused by hypertension and hyperglycemia and are not effective against fibrosis of other etiologies. Active products isolated from plants have long served as a source for drug discovery and development. Various active ingredients derived from medicinal plants have been developed for the clinical management of CVDs. For example, curcumin [17] and cryptotanshinone [18] have been reported to attenuate cardiac fibrosis. Additionally, astragaloside has been comprehensively demonstrated to target the transient receptor potential cation channel subfamily M member 7 (TRPM7)‐TGF‐*β*/Smads pathway and modulate the mitogen‐activated protein kinase (MAPK) signaling pathway, thereby significantly improving cardiac fibrosis [19–21].

By using RNA‐seq and bioinformatics analysis, we identified PYK2 as a potential key regulator involved in the occurrence and development of cardiac fibrosis. In TGF‐*β*1‐induced NIH/3T3 fibroblasts and cardiac tissues from mice with cardiac fibrosis, PYK2 was significantly increased at both transcriptional and translational levels; similarly, p‐PYK2 levels were also increased, consistent with a previous report that PYK2 activity was increased in a rat model of heart failure due to myocardial infarction [15]. PYK2 is an upstream regulator of the ERK1/2 signaling pathway. Our results demonstrated that changes in the expression and phosphorylation levels of PYK2 and p‐PYK2 were highly consistent with those of ERK1/2 and p‐ERK1/2 in both NIH/3T3 cells and cardiac fibrosis model mice induced by TGF‐*β*1. ERK1/2 is a central component of the MAPK signaling pathway, through which extracellular signals are transmitted into cells via transmembrane receptors. These signals are subsequently relayed through a cascade involving serine/threonine kinases and tyrosine kinases. Upon activation, ERK1/2 translocates from the cytoplasm to the nucleus, where it phosphorylates numerous substrates and regulates critical cellular processes including proliferation and differentiation [22]. In subsequent experiments, further investigation into intermediate cascade signaling proteins will be essential to confirm that PYK2 exerts its antifibrotic effects through regulation of the ERK signaling axis.

Liang et al. reported that LYC could inhibit pulmonary fibrosis. They predicted the interaction between LYC and the PYD domain of ASC by molecular docking and further verified this interaction between them by surface plasmon resonance technology [23]. Inspired by these findings, we will subsequently attempt to identify potential binding sites between LYC and PYK2 using molecular docking simulations. However, our experimental results demonstrated that both mRNA and protein levels of PYK2 were upregulated upon TGF‐*β*1 induction, and this increase was suppressed by pretreatment with a high dose of LYC (H). Based on these observations, we hypothesize that LYC may not bind directly to the PYK2 protein, but rather may be involved in regulating PYK2 transcription. This raises a further question: As a small molecule compound, how does LYC modulate PYK2 transcription? Through a literature search, we found that after activation, PYK2 leads to signal transducer and activator of transcription 3 (STAT3) phosphorylation through a signal cascade and phosphorylated STAT3 (p‐STAT3) translocates to the nucleus to regulate PYK2 transcription, forming a positive feedback loop [24]. As a major member of the signal transducer and activator of transcription family, STAT3 has been shown to play multiple roles in CVDs. Ye et al. found that celastrol could inhibit STAT3 function and significantly alleviate angiotensin II‐induced cardiomyocyte hypertrophy and fibrosis [25]. Whether p‐STAT3 regulates PYK2 transcription in cardiac fibrosis remains to be investigated in the future. If p‐STAT3 does enhance PYK2 transcription, the mechanism by which LYC affects STAT3 phosphorylation still needs to be elucidated.

Although our in vitro experiments demonstrated that LYC inhibits fibroblast activation, proliferation, and migration through the regulation of PYK2, whether the same PYK2‐dependent mechanism operates in vivo remains unclear in this study. In addition, we used doxorubicin (DOX) to establish a mouse model of cardiac fibrosis. The mechanisms of DOX‐induced injury involve oxidative stress, DNA damage, apoptosis, and inflammation [26–29]. However, a major limitation of this model is that its pathological characteristics differ from those of common etiologies, such as the replacement fibrosis and systolic dysfunction seen in myocardial infarction or heart failure with reduced EF. This model also lacks the comorbidities commonly observed in human diseases, such as hypertension or cancer [30, 31]. Although findings on shared mechanisms (e.g., reactive oxygen species, immune cell infiltration, and Col production) are partially applicable to broader scenarios of cardiac fibrosis, etiology‐specific variances exist. These limitations collectively constitute a critical translational gap. To bridge this gap, we plan to use cardiac fibroblast‐specific PYK2 knockout mice in a mouse model of cardiac fibrosis in our follow‐up studies, which will help determine whether the antifibrotic effect of LYC is indeed PYK2‐dependent in vivo. Furthermore, we will employ multiple cardiac fibrosis models and increase sample sizes and will also perform Col contraction assays to more comprehensively evaluate fibroblast functional changes, which will help establish whether the observed PYK2‐related mechanism is model‐independent and relevant to human disease.

## 5. Conclusion

This study provides the first evidence that LYC exerts antifibrotic effects by regulating the expression and activity of PYK2, thereby mitigating cardiac fibrosis. These findings position LYC as a promising therapeutic candidate for cardiac fibrosis, with the potential to improve cardiac function and quality of life in patients with CVDs. Furthermore, this study validates PYK2 as a viable therapeutic target and provides a rationale for the development of PYK2‐targeted inhibitors for the treatment of cardiac fibrosis.

**Nomenclature**
TGF‐*β*1: transforming growth factor‐*β*1
LYC: lycorine
DOX: doxorubicin
PYK2: proline‐rich tyrosine kinase 2
p‐PYK2: phosphorylated PYK2
ECM: extracellular matrix
*α*‐SMA: alpha‐smooth muscle actin
Col: collagen
Smads: Sma and mothers against decapentaplegic
SIRT1: silent information regulator
PPAR*γ*
: peroxisome proliferator‐activated receptor *γ*
CVDs: cardiovascular diseases
FAK: focal adhesion kinase
VEGFA: vascular endothelial growth factor
Cx43: connexin 43
Ccn2: connective tissue growth factor
Mmp 9: matrix metalloproteinase‐9
Fn1: fibronectin 1
EF: ejection fraction
FS: fractional shortening
CO: cardiac output
SV: stroke volume
cTnT: cardiac troponin T
CK‐MB: creatine kinase, Mb form
LDH: lactate dehydrogenase
DEGs: differentially expressed genes
Trpv2: transient receptor potential vanilloid ion channel subtypes 2
Itgb3: integrin subunit beta 3
Notch4: neurogenic locus notch homolog protein 4
ERK1/2: extracellular signal‐regulated kinase 1/2
P‐ERK1/2: phosphorylated ERK1/2
TRPM7: transient receptor potential cation channel subfamily M member 7
MAPK: mitogen‐activated protein kinase
STAT3: signal transducer and activator of transcription 3
p‐STAT3: phosphorylated STAT3

## Author Contributions

Xiuxiu Gao and Ying Zhang are co‐first authors. Xiuxiu Gao: writing—original draft, methodology, and validation. Ying Zhang: formal analysis, methodology, and investigation. Dongzhe Li: validation. Yufei Chen: validation. Weimiao Qi: validation. Shufang Cui: funding acquisition. Chen Wang: funding acquisition and supervision. Zhong Wang: resources and conceptualization. Quanyi Wang: writing—review and editing, funding acquisition, conceptualization, and project administration.

## Funding

The study was funded by the National Natural Science Foundation for Youth (82300337), the Jiangsu Provincial Crop Germplasm Resource Bank (Lycoris) (JS‐ZW‐K04), the Forestry Science, Technology Popularization Demonstration Project of the Central Finance (Su (2024) TG06), and the Natural Science Foundation of Jiangsu Province (BK20253030 BK20220152).

## Ethics Statement

All experimental procedures involving animals were reviewed and approved by the Institutional Animal Care and Use Committee (IACUC) of China Pharmaceutical University.

## Conflicts of Interest

The authors declare no conflicts of interest.

## Data Availability

The datasets supporting the findings of this study are available from the corresponding authors upon reasonable request.

## References

[1] Roth G. A. , Mensah G. A. , Johnson C. O. , Addolorato G. , Ammirati E. , Baddour L. M. , Barengo N. C. , Beaton A. Z. , Benjamin E. J. , Benziger C. P. , Bonny A. , Brauer M. , Brodmann M. , Cahill T. J. , Carapetis J. , Catapano A. L. , Chugh S. S. , Cooper L. T. , Coresh J. , Criqui M. , DeCleene N. , Eagle K. A. , Emmons-Bell S. , Feigin V. L. , Fernández-Solà J. , Fowkes G. , Gakidou E. , Grundy S. M. , He F. J. , Howard G. , Hu F. , Inker L. , Karthikeyan G. , Kassebaum N. , Koroshetz W. , Lavie C. , Lloyd-Jones D. , Lu H. S. , Mirijello A. , Temesgen A. M. , Mokdad A. , Moran A. E. , Muntner P. , Narula J. , Neal B. , Ntsekhe M. , Moraes de Oliveira G. , Otto C. , Owolabi M. , Pratt M. , Rajagopalan S. , Reitsma M. , Ribeiro A. L. P. , Rigotti N. , Rodgers A. , Sable C. , Shakil S. , Sliwa-Hahnle K. , Stark B. , Sundström J. , Timpel P. , Tleyjeh I. M. , Valgimigli M. , Vos T. , Whelton P. K. , Yacoub M. , Zuhlke L. , Murray C. , Fuster V. , Roth G. A. , Mensah G. A. , Johnson C. O. , Addolorato G. , Ammirati E. , Baddour L. M. , Barengo N. C. , Beaton A. , Benjamin E. J. , Benziger C. P. , Bonny A. , Brauer M. , Brodmann M. , Cahill T. J. , Carapetis J. R. , Catapano A. L. , Chugh S. , Cooper L. T. , Coresh J. , Criqui M. H. , DeCleene N. K. , Eagle K. A. , Emmons-Bell S. , Feigin V. L. , Fernández-Sola J. , Fowkes F. G. R. , Gakidou E. , Grundy S. M. , He F. J. , Howard G. , Hu F. , Inker L. , Karthikeyan G. , Kassebaum N. J. , Koroshetz W. J. , Lavie C. , Lloyd-Jones D. , Lu H. S. , Mirijello A. , Misganaw A. T. , Mokdad A. H. , Moran A. E. , Muntner P. , Narula J. , Neal B. , Ntsekhe M. , Oliveira G. M. M. , Otto C. M. , Owolabi M. O. , Pratt M. , Rajagopalan S. , Reitsma M. B. , Ribeiro A. L. P. , Rigotti N. A. , Rodgers A. , Sable C. A. , Shakil S. S. , Sliwa K. , Stark B. A. , Sundström J. , Timpel P. , Tleyjeh I. I. , Valgimigli M. , Vos T. , Whelton P. K. , Yacoub M. , Zuhlke L. J. , Abbasi-Kangevari M. , Abdi A. , Abedi A. , Aboyans V. , Abrha W. A. , Abu-Gharbieh E. , Abushouk A. I. , Acharya D. , Adair T. , Adebayo O. M. , Ademi Z. , Advani S. M. , Afshari K. , Afshin A. , Agarwal G. , Agasthi P. , Ahmad S. , Ahmadi S. , Ahmed M. B. , Aji B. , Akalu Y. , Akande-Sholabi W. , Aklilu A. , Akunna C. J. , Alahdab F. , al-Eyadhy A. , Alhabib K. F. , Alif S. M. , Alipour V. , Aljunid S. M. , Alla F. , Almasi-Hashiani A. , Almustanyir S. , al-Raddadi R. M. , Amegah A. K. , Amini S. , Aminorroaya A. , Amu H. , Amugsi D. A. , Ancuceanu R. , Anderlini D. , Andrei T. , Andrei C. L. , Ansari-Moghaddam A. , Anteneh Z. A. , Antonazzo I. C. , Antony B. , Anwer R. , Appiah L. T. , Arabloo J. , Ärnlöv J. , Artanti K. D. , Ataro Z. , Ausloos M. , Avila-Burgos L. , Awan A. T. , Awoke M. A. , Ayele H. T. , Ayza M. A. , Azari S. , Baheiraei N. , Baig A. A. , Bakhtiari A. , Banach M. , Banik P. C. , Baptista E. A. , Barboza M. A. , Barua L. , Basu S. , Bedi N. , Béjot Y. , Bennett D. A. , Bensenor I. M. , Berman A. E. , Bezabih Y. M. , Bhagavathula A. S. , Bhaskar S. , Bhattacharyya K. , Bijani A. , Bikbov B. , Birhanu M. M. , Boloor A. , Brant L. C. , Brenner H. , Briko N. I. , Butt Z. A. , Caetano dos Santos F. L. , Cahill L. E. , Cahuana-Hurtado L. , Cámera L. A. , Campos-Nonato I. R. , Cantu-Brito C. , Car J. , Carrero J. J. , Carvalho F. , Castañeda-Orjuela C. A. , Catalá-López F. , Cerin E. , Charan J. , Chattu V. K. , Chen S. , Chin K. L. , Choi J. Y. J. , Chu D. T. , Chung S. C. , Cirillo M. , Coffey S. , Conti S. , Costa V. M. , Cundiff D. K. , Dadras O. , Dagnew B. , Dai X. , Damasceno A. A. M. , Dandona L. , Dandona R. , Davletov K. , de la Cruz-Góngora V. , de la Hoz F. P. , de Neve J. W. , Denova-Gutiérrez E. , Derbew Molla M. , Derseh B. T. , Desai R. , Deuschl G. , Dharmaratne S. D. , Dhimal M. , Dhungana R. R. , Dianatinasab M. , Diaz D. , Djalalinia S. , Dokova K. , Douiri A. , Duncan B. B. , Duraes A. R. , Eagan A. W. , Ebtehaj S. , Eftekhari A. , Eftekharzadeh S. , Ekholuenetale M. , el Nahas N. , Elgendy I. Y. , Elhadi M. , el-Jaafary S. I. , Esteghamati S. , Etisso A. E. , Eyawo O. , Fadhil I. , Faraon E. J. A. , Faris P. S. , Farwati M. , Farzadfar F. , Fernandes E. , Fernandez Prendes C. , Ferrara P. , Filip I. , Fischer F. , Flood D. , Fukumoto T. , Gad M. M. , Gaidhane S. , Ganji M. , Garg J. , Gebre A. K. , Gebregiorgis B. G. , Gebregzabiher K. Z. , Gebremeskel G. G. , Getacher L. , Obsa A. G. , Ghajar A. , Ghashghaee A. , Ghith N. , Giampaoli S. , Gilani S. A. , Gill P. S. , Gillum R. F. , Glushkova E. V. , Gnedovskaya E. V. , Golechha M. , Gonfa K. B. , Goudarzian A. H. , Goulart A. C. , Guadamuz J. S. , Guha A. , Guo Y. , Gupta R. , Hachinski V. , Hafezi-Nejad N. , Haile T. G. , Hamadeh R. R. , Hamidi S. , Hankey G. J. , Hargono A. , Hartono R. K. , Hashemian M. , Hashi A. , Hassan S. , Hassen H. Y. , Havmoeller R. J. , Hay S. I. , Hayat K. , Heidari G. , Herteliu C. , Holla R. , Hosseini M. , Hosseinzadeh M. , Hostiuc M. , Hostiuc S. , Househ M. , Huang J. , Humayun A. , Iavicoli I. , Ibeneme C. U. , Ibitoye S. E. , Ilesanmi O. S. , Ilic I. M. , Ilic M. D. , Iqbal U. , Irvani S. S. N. , Islam S. M. S. , Islam R. M. , Iso H. , Iwagami M. , Jain V. , Javaheri T. , Jayapal S. K. , Jayaram S. , Jayawardena R. , Jeemon P. , Jha R. P. , Jonas J. B. , Jonnagaddala J. , Joukar F. , Jozwiak J. J. , Jürisson M. , Kabir A. , Kahlon T. , Kalani R. , Kalhor R. , Kamath A. , Kamel I. , Kandel H. , Kandel A. , Karch A. , Kasa A. S. , Katoto P. D. M. C. , Kayode G. A. , Khader Y. S. , Khammarnia M. , Khan M. S. , Khan M. N. , Khan M. , Khan E. A. , Khatab K. , Kibria G. M. A. , Kim Y. J. , Kim G. R. , Kimokoti R. W. , Kisa S. , Kisa A. , Kivimäki M. , Kolte D. , Koolivand A. , Korshunov V. A. , Koulmane Laxminarayana S. L. , Koyanagi A. , Krishan K. , Krishnamoorthy V. , Kuate Defo B. , Kucuk Bicer B. , Kulkarni V. , Kumar G. A. , Kumar N. , Kurmi O. P. , Kusuma D. , Kwan G. F. , la Vecchia C. , Lacey B. , Lallukka T. , Lan Q. , Lasrado S. , Lassi Z. S. , Lauriola P. , Lawrence W. R. , Laxmaiah A. , LeGrand K. E. , Li M. C. , Li B. , Li S. , Lim S. S. , Lim L. L. , Lin H. , Lin Z. , Lin R. T. , Liu X. , Lopez A. D. , Lorkowski S. , Lotufo P. A. , Lugo A. , Madotto F. , Mahmoudi M. , Majeed A. , Malekzadeh R. , Malik A. A. , Mamun A. A. , Manafi N. , Mansournia M. A. , Mantovani L. G. , Martini S. , Mathur M. R. , Mazzaglia G. , Mehata S. , Mehndiratta M. M. , Meier T. , Menezes R. G. , Meretoja A. , Mestrovic T. , Miazgowski B. , Miazgowski T. , Michalek I. M. , Miller T. R. , Mirrakhimov E. M. , Mirzaei H. , Moazen B. , Moghadaszadeh M. , Mohammad Y. , Mohammad D. K. , Mohammed S. , Mohammed M. A. , Mokhayeri Y. , Molokhia M. , Montasir A. A. , Moradi G. , Moradzadeh R. , Moraga P. , Morawska L. , Moreno Velásquez I. , Morze J. , Mubarik S. , Muruet W. , Musa K. I. , Nagarajan A. J. , Nalini M. , Nangia V. , Naqvi A. A. , Narasimha Swamy S. , Nascimento B. R. , Nayak V. C. , Nazari J. , Nazarzadeh M. , Negoi R. I. , Neupane Kandel S. , Nguyen H. L. T. , Nixon M. R. , Norrving B. , Noubiap J. J. , Nouthe B. E. , Nowak C. , Odukoya O. O. , Ogbo F. A. , Olagunju A. T. , Orru H. , Ortiz A. , Ostroff S. M. , Padubidri J. R. , Palladino R. , Pana A. , Panda-Jonas S. , Parekh U. , Park E. C. , Parvizi M. , Pashazadeh Kan F. , Patel U. K. , Pathak M. , Paudel R. , Pepito V. C. F. , Perianayagam A. , Perico N. , Pham H. Q. , Pilgrim T. , Piradov M. A. , Pishgar F. , Podder V. , Polibin R. V. , Pourshams A. , Pribadi D. R. A. , Rabiee N. , Rabiee M. , Radfar A. , Rafiei A. , Rahim F. , Rahimi-Movaghar V. , Ur Rahman M. H. , Rahman M. A. , Rahmani A. M. , Rakovac I. , Ram P. , Ramalingam S. , Rana J. , Ranasinghe P. , Rao S. J. , Rathi P. , Rawal L. , Rawasia W. F. , Rawassizadeh R. , Remuzzi G. , Renzaho A. M. N. , Rezapour A. , Riahi S. M. , Roberts-Thomson R. L. , Roever L. , Rohloff P. , Romoli M. , Roshandel G. , Rwegerera G. M. , Saadatagah S. , Saber-Ayad M. M. , Sabour S. , Sacco S. , Sadeghi M. , Saeedi Moghaddam S. , Safari S. , Sahebkar A. , Salehi S. , Salimzadeh H. , Samaei M. , Samy A. M. , Santos I. S. , Santric-Milicevic M. M. , Sarrafzadegan N. , Sarveazad A. , Sathish T. , Sawhney M. , Saylan M. , Schmidt M. I. , Schutte A. E. , Senthilkumaran S. , Sepanlou S. G. , Sha F. , Shahabi S. , Shahid I. , Shaikh M. A. , Shamali M. , Shamsizadeh M. , Shawon M. S. R. , Sheikh A. , Shigematsu M. , Shin M. J. , Shin J. I. , Shiri R. , Shiue I. , Shuval K. , Siabani S. , Siddiqi T. J. , Silva D. A. S. , Singh J. A. , Mtech A. S. , Skryabin V. Y. , Skryabina A. A. , Soheili A. , Spurlock E. E. , Stockfelt L. , Stortecky S. , Stranges S. , Suliankatchi Abdulkader R. , Tadbiri H. , Tadesse E. G. , Tadesse D. B. , Tajdini M. , Tariqujjaman M. , Teklehaimanot B. F. , Temsah M. H. , Tesema A. K. , Thakur B. , Thankappan K. R. , Thapar R. , Thrift A. G. , Timalsina B. , Tonelli M. , Touvier M. , Tovani-Palone M. R. , and Tripathi A. , Global Burden of Cardiovascular Diseases and Risk Factors, 1990-2019: Update From the GBD 2019 Study, Journal of the American College of Cardiology. (2020) 76, no. 25, 2982–3021, 10.1016/j.jacc.2020.11.010, 33309175. 33309175 PMC7755038

[2] Liu M. , He X. , Yang X. , and Wang Z. , 2024 Annual Report on Cardiovascular Health and Diseases in China: Data and Trend, Chinese Medical Journal. (2025) 138, no. 23, 3037–3049, 10.1097/cm9.0000000000003902, 41234018. 41234018 PMC12700738

[3] van den Borne S. W. M. , Diez J. , Blankesteijn W. M. , Verjans J. , Hofstra L. , and Narula J. , Myocardial Remodeling After Infarction: The Role of Myofibroblasts, Nature Reviews Cardiology. (2010) 7, no. 1, 30–37, 10.1038/nrcardio.2009.199. 19949426

[4] Goumans M. J. and Ten Dijke P. , TGF-*β* Signaling in Control of Cardiovascular Function, CSH Perspectives in Biology. (2018) 10, no. 2, 10.1101/cshperspect.a022210, 28348036. PMC5793760 28348036

[5] Tuo P. , Zhao R. , Li N. , Yan S. , Yang G. , Wang C. , Sun J. , Sun H. , and Wang M. , Lycorine Inhibits Ang II-Induced Heart Remodeling and Inflammation by Suppressing the PI3K-AKT/NF-*κ*B Pathway, Phytomedicine. (2024) 128, 155464, 10.1016/j.phymed.2024.155464, 38484625. 38484625

[6] Wu J. , Fu Y. , Wu Y. X. , Wu Z. X. , Wang Z. H. , and Li P. , Lycorine Ameliorates Isoproterenol-Induced Cardiac Dysfunction Mainly Via Inhibiting Inflammation, Fibrosis, Oxidative Stress And Apoptosis, Bioengineered. (2021) 12, no. 1, 5583–5594, 10.1080/21655979.2021.1967019, 34515620. 34515620 PMC8806515

[7] Zhou Q. , Jin X. , Yuan M. , Tao H. , Bao S. , Zhang J. , Xu Z. , Chattipakorn N. , Wang Y. , Zhu H. , and Liang G. , Lycorine Improves Inflammatory Imbalance in Diabetic Cardiomyopathy by Targeting ILF3, Phytomedicine. (2026) 153, 157976, 10.1016/j.phymed.2026.157976, 41720008. 41720008

[8] Schimmel K. , Jung M. , Foinquinos A. , José G. S. , Beaumont J. , Bock K. , Grote-Levi L. , Xiao K. , Bär C. , Pfanne A. , Just A. , Zimmer K. , Ngoy S. , López B. , Ravassa S. , Samolovac S. , Janssen-Peters H. , Remke J. , Scherf K. , Dangwal S. , Piccoli M. T. , Kleemiss F. , Kreutzer F. P. , Kenneweg F. , Leonardy J. , Hobuß L. , Santer L. , do Q. T. , Geffers R. , Braesen J. H. , Schmitz J. , Brandenberger C. , Müller D. N. , Wilck N. , Kaever V. , Bähre H. , Batkai S. , Fiedler J. , Alexander K. M. , Wertheim B. M. , Fisch S. , Liao R. , Diez J. , González A. , and Thum T. , Natural Compound Library Screening Identifies New Molecules for the Treatment of Cardiac Fibrosis and Diastolic Dysfunction, Circulation. (2020) 141, no. 9, 751–767, 10.1161/circulationaha.119.042559, 31948273. 31948273 PMC7050799

[9] Wang Z. , Chen Y. , Gu M. , Wu Z. , Ding B. , Yang W. , Wu X. , Wang C. , Gao X. , Yang Y. , and Yin G. , Protective Effects and Mechanisms of Lycorine Against Adriamycin-Induced Cardiotoxicity, Phytomedicine. (2022) 102, 154178, 10.1016/j.phymed.2022.154178, 35617889. 35617889

[10] Zhao H. , Chen Y. , Qian L. , Du L. , Wu X. , Tian Y. , Deng C. , Liu S. , Yang W. , Lu C. , Zhang Y. , Ren J. , and Yang Y. , Lycorine Protects Against Septic Myocardial Injury by Activating AMPK-Related Pathways, Free Radical Biology and Medicine. (2023) 197, 1–14, 10.1016/j.freeradbiomed.2023.01.010, 36669544. 36669544

[11] Ivaska J. and Heino J. , Cooperation Between Integrins and Growth Factor Receptors in Signaling and Endocytosis, Annual Review of Cell and Developmental Biology. (2011) 27, no. 1, 291–320, 10.1146/annurev-cellbio-092910-154017, 21663443. 21663443

[12] Guan X. , Liu Y. , An Y. , Wang X. , Wei L. , and Qi X. , FAK Family Kinases: A Potential Therapeutic Target for Atherosclerosis, Diabetes, Metabolic Syndrome and Obesity. (2024) 17, 3151–3161, 10.2147/dmso.S465755, 39220801. PMC11363942 39220801

[13] Murphy J. M. , Jeong K. , and Lim S. S. , FAK Family Kinases in Vascular Diseases, International Journal of Molecular Sciences. (2020) 21, no. 10, 10.3390/ijms21103630, 32455571. PMC7279255 32455571

[14] Murphy J. M. , Jeong K. , Rodriguez Y. A. R. , Kim J. H. , Ahn E. E. , and Lim S. S. , FAK and Pyk2 Activity Promote TNF-*α* and IL-1*β*-Mediated Pro-Inflammatory Gene Expression and Vascular Inflammation, Scientific Reports. (2019) 9, no. 1, 10.1038/s41598-019-44098-2, 31110200. PMC6527705 31110200

[15] Zheng L. , Spagnol G. , Gandhi D. R. , Sharma K. , Kumar V. , Patel K. P. , and Sorgen P. L. , Inhibition of Pyk2 Improves Cx43 Intercalated Disc Localization, Infarct Size, and Cardiac Function in Rats With Heart Failure, Circulation: Heart Failure. (2023) 16, no. 8, e010294, 10.1161/circheartfailure.122.010294, 37465947. 37465947 PMC10524803

[16] Zheng L. , Trease A. J. , Katsurada K. , Spagnol G. , Li H. , Shi W. , Duan B. , Patel K. P. , and Sorgen P. L. , Inhibition of Pyk2 and Src Activity Improves Cx43 Gap Junction Intercellular Communication, Journal of Molecular and Cellular Cardiology. (2020) 149, 27–40, 10.1016/j.yjmcc.2020.09.004, 32956670. 32956670 PMC7736531

[17] Yang C. , Zhu Q. , Chen Y. , Ji K. , Li S. , Wu Q. , Pan Q. , and Li J. , Review of the Protective Mechanism of Curcumin on Cardiovascular Disease, Drug Design, Development and Therapy. (2024) 18, 165–192, 10.2147/dddt.S445555, 38312990. 38312990 PMC10838105

[18] Ma Y. , Li H. , Yue Z. , Guo J. , Xu S. , Xu J. , Jia Y. , Yu N. , Zhang B. , Liu S. , Liu M. , Shao W. , Chen S. , and Liu P. , Cryptotanshinone Attenuates Cardiac Fibrosis via Downregulation of COX-2, NOX-2, and NOX-4, Journal of Cardiovascular Pharmacology. (2014) 64, no. 1, 28–37, 10.1097/fjc.0000000000000086, 24621647. 24621647

[19] Lu J. , Wang Q. Y. , Zhou Y. , Lu X. C. , Liu Y. H. , Wu Y. , Guo Q. , Ma Y. T. , and Tang Y. Q. , AstragalosideIV Against Cardiac Fibrosis by Inhibiting TRPM7 Channel, Phytomedicine. (2017) 30, 10–17, 10.1016/j.phymed.2017.04.002, 28545665. 28545665

[20] Sun C. , Zeng G. , Wang T. , Ren H. , An H. , Lian C. , Liu J. , Guo L. , and Li W. , Astragaloside IV Ameliorates Myocardial Infarction Induced Apoptosis and Restores Cardiac Function, Frontiers in Cell and Developmental Biology. (2021) 9, 671255, 10.3389/fcell.2021.671255, 34395418. 34395418 PMC8358605

[21] Wei Y. , Wu Y. , Feng K. , Zhao Y. , Tao R. , Xu H. , and Tang Y. , Astragaloside IV Inhibits Cardiac Fibrosis via miR-135a-TRPM7-TGF-*β*/Smads Pathway, Journal of Ethnopharmacology. (2020) 249, 112404, 10.1016/j.jep.2019.112404, 31739105. 31739105

[22] Gallo S. , Vitacolonna A. , Bonzano A. , Comoglio P. , and Crepaldi T. , ERK: A Key Player in the Pathophysiology of Cardiac Hypertrophy, Int J Mol Sci. (2019) 20, no. 9, 10.3390/ijms20092164, 31052420. PMC6539093 31052420

[23] Liang Q. , Cai W. , Zhao Y. , Xu H. , Tang H. , Chen D. , Qian F. , and Sun L. , Lycorine Ameliorates Bleomycin-Induced Pulmonary Fibrosis via Inhibiting NLRP3 Inflammasome Activation and Pyroptosis, Pharmacological Research. (2020) 158, 104884, 10.1016/j.phrs.2020.104884, 32428667. 32428667

[24] Gil-Henn H. , Girault J. A. , and Lev S. , PYK2, a Hub of Signaling Networks in Breast Cancer Progression, Trends in Cell Biology. (2024) 34, no. 4, 312–326, 10.1016/j.tcb.2023.07.006, 37586982. 37586982

[25] Ye S. , Luo W. , Khan Z. A. , Wu G. , Xuan L. , Shan P. , Lin K. , Chen T. , Wang J. , Hu X. , Wang S. , Huang W. , and Liang G. , Celastrol Attenuates Angiotensin II-Induced Cardiac Remodeling by Targeting STAT3, Circulation Research. (2020) 126, no. 8, 1007–1023, 10.1161/circresaha.119.315861, 32098592. 32098592

[26] Arrigoni R. , Jirillo E. , and Caiati C. , Pathophysiology of Doxorubicin-Mediated Cardiotoxicity, Toxics. (2025) 13, no. 4, 10.3390/toxics13040277, 40278593. PMC12031459 40278593

[27] Levick S. P. , Soto-Pantoja D. R. , Bi J. , Hundley W. G. , Widiapradja A. , Manteufel E. J. , Bradshaw T. W. , and Meléndez G. C. , Doxorubicin-Induced Myocardial Fibrosis Involves the Neurokinin-1 Receptor and Direct Effects on Cardiac Fibroblasts, Heart, Lung and Circulation. (2019) 28, no. 10, 1598–1605, 10.1016/j.hlc.2018.08.003, 30205930. PMC7901001 30205930

[28] Podyacheva E. , Shmakova T. , Kushnareva E. , Onopchenko A. , Martynov M. , Andreeva D. , Toropov R. , Cheburkin Y. , Levchuk K. , Goldaeva A. , and Toropova Y. , Modeling Doxorubicin-Induced Cardiomyopathy With Fibrotic Myocardial Damage in Wistar Rats, Cardiology Research. (2022) 13, no. 6, 339–356, 10.14740/cr1416, 36660062. 36660062 PMC9822674

[29] Podyacheva E. Y. , Kushnareva E. A. , Karpov A. A. , and Toropova Y. G. , Analysis of Models of Doxorubicin-Induced Cardiomyopathy in Rats and Mice. A Modern View From the Perspective of the Pathophysiologist and the Clinician, Frontiers in Pharmacology. (2021) 12, 670479, 10.3389/fphar.2021.670479, 34149423. 34149423 PMC8209419

[30] Linders A. N. , Dias I. B. , López Fernández T. , Tocchetti C. G. , Bomer N. , and Van der Meer P. , A Review of the Pathophysiological Mechanisms of Doxorubicin-Induced Cardiotoxicity and Aging, NPJ Aging. (2024) 10, no. 1, 10.1038/s41514-024-00135-7, 38263284. PMC10806194 38263284

[31] Zhu W. , Shou W. , Payne R. M. , Caldwell R. , and Field L. J. , A Mouse Model for Juvenile Doxorubicin-Induced Cardiac Dysfunction, Pediatric Research. (2008) 64, no. 5, 488–494, 10.1203/PDR.0b013e318184d732, 18614963. 18614963 PMC2801890

